# Multiple Routes to Animal Consciousness: Constrained Multiple Realizability Rather Than Modest Identity Theory

**DOI:** 10.3389/fpsyg.2021.732336

**Published:** 2021-09-24

**Authors:** Jon Mallatt, Todd E. Feinberg

**Affiliations:** ^1^The University of Washington WWAMI Medical Education Program at The University of Idaho, Moscow, ID, United States; ^2^Department of Psychiatry and Neurology, Icahn School of Medicine at Mount Sinai, New York, NY, United States

**Keywords:** animal consciousness, multiple realizability, convergent evolution, mental constraint thesis, modest identity thesis, compensatory differences, mental phenomena, evolutionary constraints

## Abstract

The multiple realizability thesis (MRT) is an important philosophical and psychological concept. It says any mental state can be constructed by multiple realizability (MR), meaning in many distinct ways from different physical parts. The goal of our study is to find if the MRT applies to the mental state of consciousness among animals. Many things have been written about MRT but the ones most applicable to animal consciousness are by Shapiro in a 2004 book called The Mind Incarnate and by Polger and Shapiro in their 2016 work, The Multiple Realization Book. Standard, classical MRT has been around since 1967 and it says that a mental state can have *very many* different physical realizations, in a nearly unlimited manner. To the contrary, Shapiro’s book reasoned that physical, physiological, and historical constraints force mental traits to evolve in just a few, limited directions, which is seen as convergent evolution of the associated neural traits in different animal lineages. This is his mental constraint thesis (MCT). We examined the evolution of consciousness in animals and found that it arose independently in just three animal clades—vertebrates, arthropods, and cephalopod mollusks—all of which share many consciousness-associated traits: elaborate sensory organs and brains, high capacity for memory, directed mobility, etc. These three constrained, convergently evolved routes to consciousness fit Shapiro’s original MCT. More recently, Polger and Shapiro’s book presented much the same thesis but changed its name from MCT to a “modest identity thesis.” Furthermore, they argued against almost all the classically offered instances of MR in animal evolution, especially against the evidence of neural plasticity and the differently expanded cerebrums of mammals and birds. In contrast, we argue that some of these classical examples of MR are indeed valid and that Shapiro’s original MCT correction of MRT is the better account of the evolution of consciousness in animal clades. And we still agree that constraints and convergence refute the standard, nearly unconstrained, MRT.

## Introduction

Our research program focuses on which animals have at least a minimal or primary form of consciousness; that is, have raw, nonreflective experiences of images constructed from sensing the world and also experience affects, meaning emotions, and moods (Mallatt and Feinberg, 2020; Mallatt et al., 2021). We have worked together on this program for almost a decade (Feinberg and Mallatt, 2013, 2016, 2018, 2019, 2020). In our work, we use systems theory to argue that consciousness is an evolved product of complex brains in complex bodies, so it is an emergent feature of a complex physical system (Feinberg and Mallatt, 2020). One feature of every emergent, complex system is that its end-process can be caused in multiple ways or by “multiple routes” (reviewed by Feinberg and Mallatt, 2020). Examples of this multiple-routes feature are: waves emerging in a body of water, which can be caused by either the wind, a stone, or an earthquake; a traffic jam, which can be caused by bad weather, too many vehicles on the road, or an accident ahead; and the patterns formed by “cellular automata,” which are computer simulations programed to follow various rules (Bedau, 2008, p. 180).

In the study of the mind, this multiple-routes feature has been called *multiple realizability (MR)*, as promoted by the *multiple realizability thesis (MRT)*, which says that a mind and its mental states can be constructed in many distinct ways from different physical parts (Bickle, 2020).^1^^,^^2^ MRT is of philosophical importance for addressing the mind-body problem because it is at the core of the dominant philosophical view called non-reductive physicalism, which says that mental states have strictly physical causes but do not reduce to counterparts in the more basic sciences, such as physics and neurobiology (Kim, 2008; Macdonald and Macdonald, 2019). Here, we emphasize the *multiple* in multiple realizability. Indeed, as Bickle (2020) points out, the most popular versions of MRT, named the “standard” and “radical” versions, say that *very many* types of physical states can cause, or realize, the same mental state.

MRT was constructed (Putnam, 1967) to refute the mind-brain *identity theory*, which says that all mental states are identical to brain states, and which itself arose as a solution to the mystery of how the mind relates to the brain (Place, 1956; Smart, 1959). The logic by which MRT argues against the identity theory is that if a mental state has many different causes; then, it has no single cause so we cannot look for any identity or even generality among the causes of the state. Each instance could have a different cause, with the causes having no physical properties in common (Baysan, 2019; Bickle, 2020).

MRT asserts—and we agree—that mental phenomena or states really do exist as mental kinds. That is, in accordance with the disciplines of psychology and neuroscience, MRT recognizes such general kinds as explicit memory, feeling acute pain, associative learning, cognitive problem solving, and consciousness, with each kind occurring as the same thing in different humans and different species. Calling these things “kinds” can always be opposed, philosophically, by successive “kind splitting” (Aizawa, 2013; Polger and Shapiro, 2016, pp. 99–104), where the opponent argues that the claimed mental state (sharp pain or memory, for example) is not the same in a rat as in a human, in a monkey as in a human, in two different humans, or in the same human at two different times. As evolutionary biologists, we resist such kind-splitting on the grounds that the mental kinds have adaptive value in multiple taxa of brainy animals. We reason that strong selection pressures demand the psychological states be the same for the different taxa to survive in competition in the same, real world. Any competing taxon without memory or attention skills would quickly go extinct.

We definitely include consciousness among the mental kinds that are shared by different taxa (Ben-Haim et al., 2021). The evidence for this that impressed us most was from Neider et al. (2020), in which crows demonstrated human-derived markers of consciousness (single-neuron responses that mark visual perception) at the same time these crows showed monkey-like cognitive skills (the ability to report their perceptions). This was good evidence for the conscious mental kind across the distantly related birds and mammals.

The present paper focuses on the studies of Lawrence Shapiro and Thomas Polger because they are the authors in the MR field who most closely considered the mental states of animals—animal consciousness being the theme of this special issue. Shapiro chose not to include consciousness among the states he analyzed for multiple realizability because consciousness has difficult, subjective aspects (Shapiro, 2004, pp. 70, 228–229). However, we see consciousness as a valid mental state that has been defined well enough and can be studied analytically and scientifically (Nagel, 1974; Mallatt and Feinberg, 2020; Mallatt et al., 2021; Mallatt, 2021a). Therefore, we will go ahead and analyze whether it is a multiply-realized phenomenon in the animal kingdom. That is the goal of this paper.

## Part 1: Shapiro on Multiple Realization and Constraints

### The Importance of Evolutionary Constraints

Because MRT claims that so many different physical mechanisms can give rise to each mental state, Shapiro investigated whether this claim fits biological reality. If, as MRT asserts, the same state has little in common across the animal taxa in its causal mechanisms, then “we should be able to make few predictions about the properties of the organ that realizes” a mental state (Shapiro, 2004, p. 137). Next, Shapiro continues, MRT claims that the functions of any state place few constraints on the properties that can cause such a state. With so few constraints, therefore, MRT also predicts there will be no or little convergent evolution of the structures related to any mental kind across distantly related taxa. To the contrary, convergent evolution is common (Conway Morris, 2003; McGhee, 2019), and Shapiro refuted these MRT predictions by documenting many examples of it, as channeled by physical, physiological, and historical constraints (also see Vogel, 1998). Shapiro’s best examples are convergently evolved similarities in different eyes and the independent evolution of modular subparts in the brains of different animals. (We document these below.)

**Figure 1:**
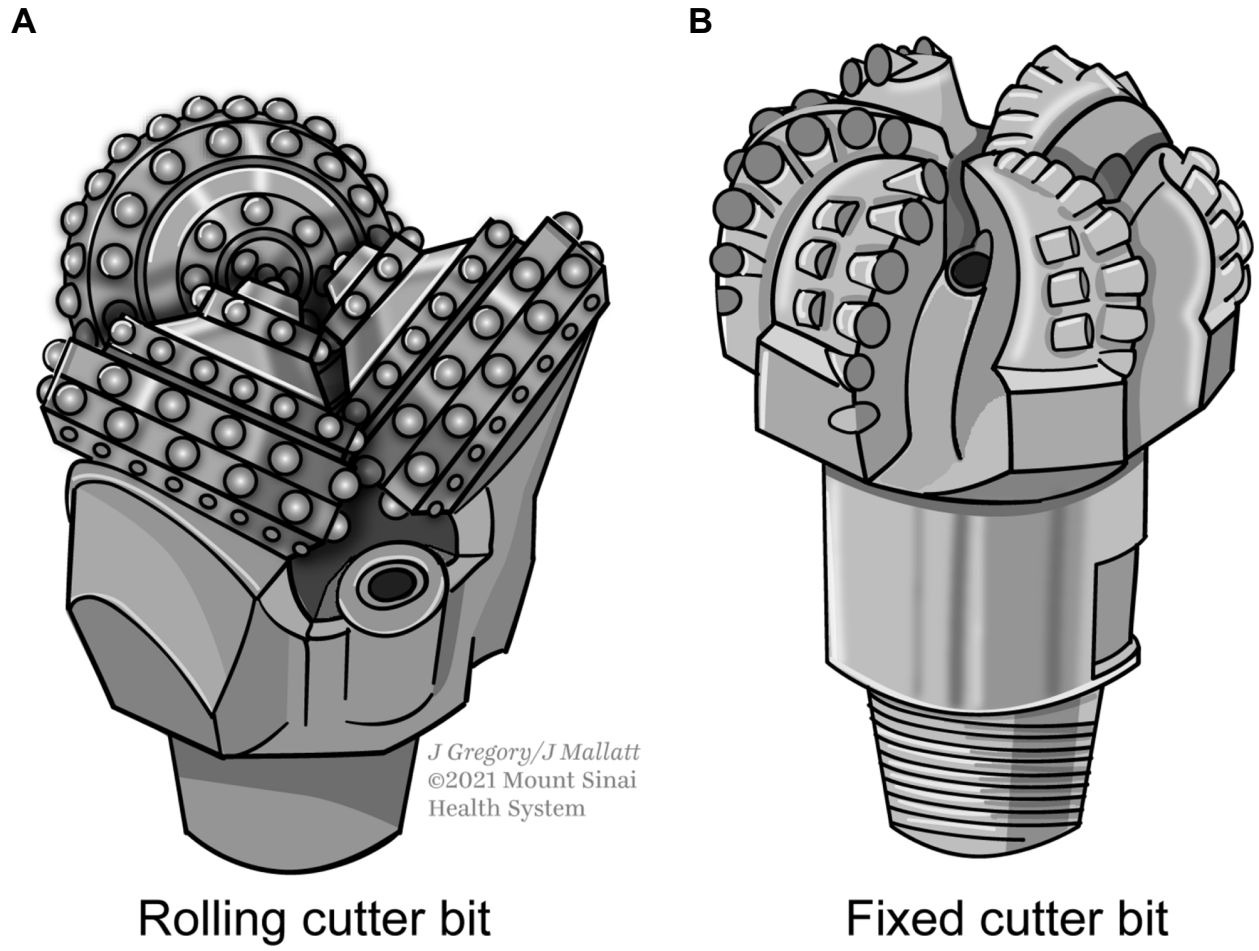
Constraints in the design of rock-drilling bits for the petroleum industry. Only two types **(A)** and **(B)** are practical for this purpose. For photos, see https://petgeo.weebly.com/types-of-drilling-bits.html.

The many documented instances where constraints produced convergent evolution led Shapiro to reject MRT as wrong for claiming that “almost anything goes.” He replaced MRT with his mental constraint thesis (MCT). This thesis says a given mental state can have only a few types of neural causes (realizers)—far fewer than allowed by standard MRT, a “handful” rather than “hundreds or thousands” (p. 32). He illustrated MCT with helpful analogies. Mechanical devices for removing the cork from a wine bottle (pp. 1–2, 46–51, 68) are constrained to those that pull, suck, push, or twist out the cork, because not much else will work. A bit that drills through rocks for oil can only consist of diamond or hardened metal and it invariably uses a rotatory action. Without these constraints, the bit could not penetrate the rock fast enough or would wear out too soon. Only two types of bits fit the necessary conditions: the rolling cutter bit and the fixed cutter bit (Figure 1).

**Figure 2:**
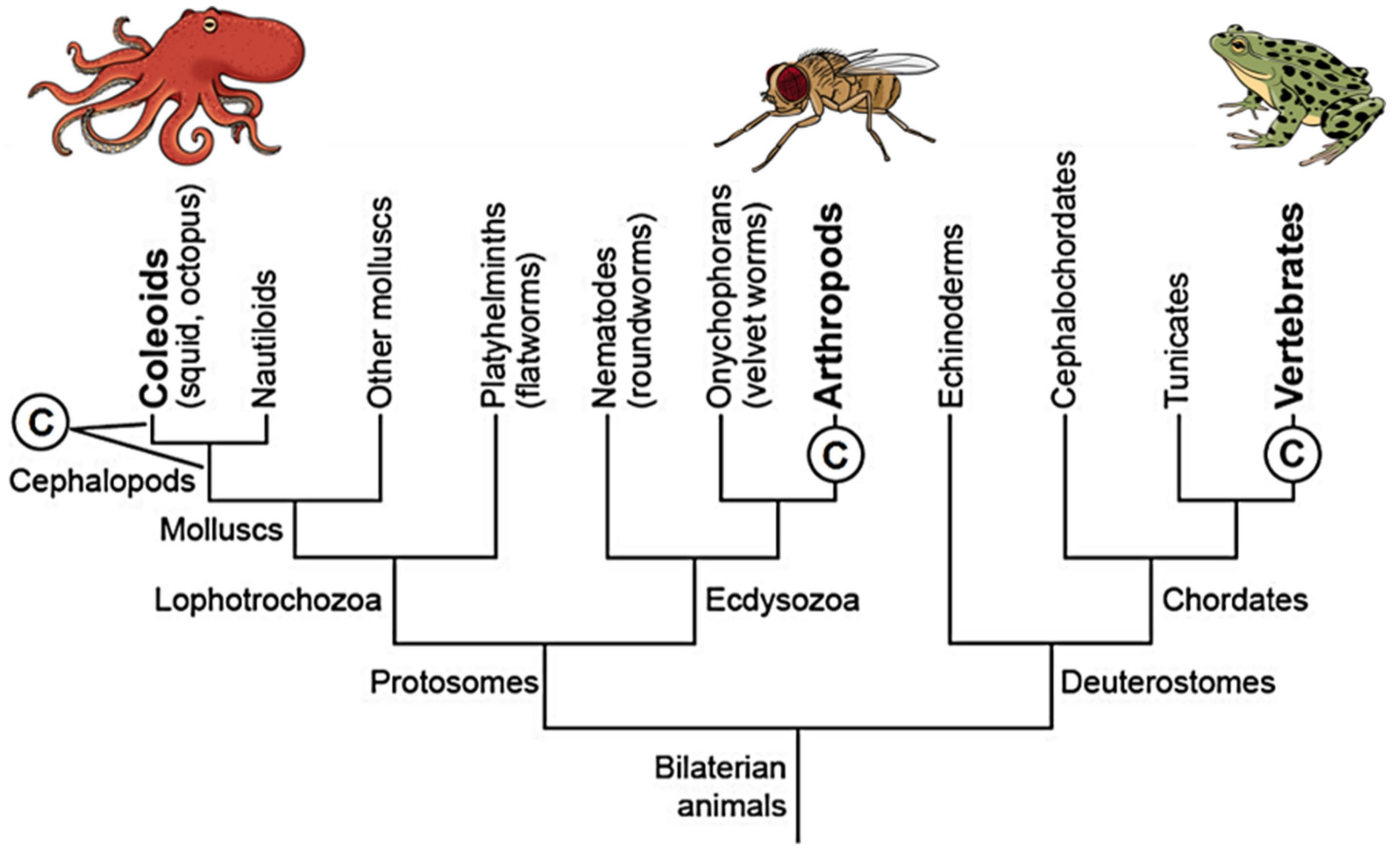
A simplified phylogenetic tree of animal relationships showing that consciousness (©) emerged independently in three different lines of animals. At left, the two leaders extending from the © mean that we cannot tell whether the consciousness evolved in the first cephalopod mollusks or else in the coleoid ancestor of squid, octopus, and cuttlefish. Reproduced with the permission of the copyright holder Mount Sinai Health System.

Does the mental state of primary consciousness fit MRT or does it fit MCT? To answer this, we must provide some background. In our prior studies, we deduced that only three clades of animals are conscious (Feinberg and Mallatt, 2016, 2018; Mallatt, 2021a,b). This deduction came from two reasoned assumptions: (1) an animal has consciousness if it builds detailed, multisensory representations of the world with mapped, topographically arranged neural pathways to and in its brain and (2) if it is capable of elaborate operant learning from rewards and punishments.^3^ The only animals that fit these criteria are all the vertebrates, all the arthropods, and cephalopod mollusks (octopus, squid, and cuttlefish). Importantly, these unrelated taxa share many consciousness-related features, which are listed in Table 1. The small number of conscious taxa—just three—indicates evolutionary convergence with constraints, MCT not MRT. We emphasize that the vertebrates, arthropods, and cephalopods fully fit the criteria for convergently evolved consciousnesses, having descended independently from a distant common ancestor that lacked a brain and was without consciousness (Northcutt, 2012; Feinberg and Mallatt, 2016; Figure 2).

**Table 1:** The convergently evolved features of consciousness that are shared by vertebrates, arthropods, and cephalopod mollusks (mostly from Feinberg and Mallatt, 2020).

Neural complexity (more than in a simple, core brain)
Brain with many neurons (>100,000?)
Many subtypes of neurons
Elaborated sensory organs
Image-forming eyes, receptors for touch, taste, hearing, smell
Neural hierarchies with neuron–neuron interactions
Extensive reciprocal communication in and between pathways for the different senses
Brain’s neural computing modules and networks are distributed but integrated, leading to local functional isolation plus global coherence
Synchronized communication by brain-wave oscillations
Neural spike trains form representational codes
The higher brain levels allow the complex processing and unity of consciousness
Higher brain levels exert considerable influence on the lower levels such as motor neurons, for top-down causality
Hierarchies that let consciousness predict events a fraction of a second in advance
Pathways that create mapped mental images or affects (affects being emotions and moods)
Neurons are arranged in topographic maps of the outside world and body structures
Valence coding of good and bad, for affective states
Feed into pre-motor brain regions to motivate, choose, and guide movements in space for high mobility
Brain mechanisms for selective attention and arousal
Memory of perceived objects or events

The fact that the MRT did not consider the constraints or the limitations that these constraints impose seems a major blind spot of that thesis. As Shapiro (2004, p. 21–23) pointed out, constraints are faced by every living system that has goal-directed functions because unless such a system is constructed in a certain, constrained way it cannot perform its function. Consciousness certainly meets this criterion of having an adaptive function that benefits survival (Cabanac, 1996; Seth, 2009; Feinberg and Mallatt, 2018), its function being to aid decision making by allowing one to consider alternate choices. Stated another way, consciousness processes complex sensory information to choose and direct the movements of large, multicellular bodies in space, for finding food and mates and for escaping danger (Table 2). The constraints necessary for this function are needing neurons, sensory organs, muscles, and many more.

**Table 2:** Some adaptive roles of consciousness (from Feinberg and Mallatt, 2019) that constrain the types of features that can produce this phenomenon.

• Consciousness organizes large amounts of sensory input into a set of phenomenal properties for choosing which actions to perform
• Its unified simulation of the sensed world directs behavior in this world
• It ranks sensed stimuli by importance, by assigning affects to them, making decisions easier (Cabanac, 1996)
• Allows flexible behavior because it sets up many different behavioral choices
• Allows easily adjustable behavior because it predicts the consequences of one’s actions into the immediate future (Perry and Chittka, 2019; Solms, 2019)
• Deals well with new situations, to meet the changing challenges of complex environments

**Figure 3:**
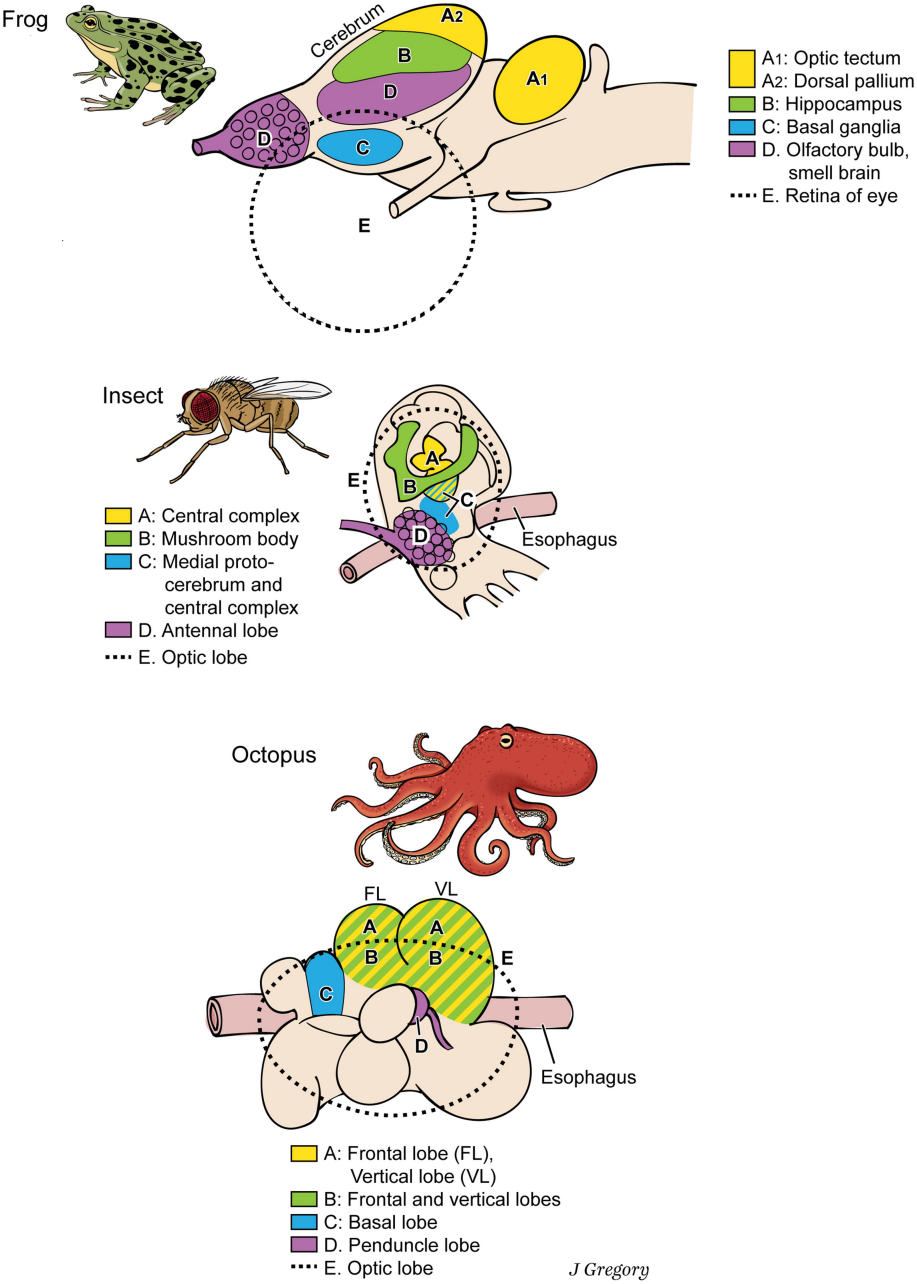
Dissimilar brains of three different taxa of animals with consciousness. The areas with similar functions are colored the same in the different brains. The general code is: **(A)**, image-based consciousness; **(B)**, memory; **(C)**, pre-motor center; **(D)**, smell processing; and **(E)**, visual processing. From *Consciousness Demystified*, MIT, 2018, reproduced with the permission of the copyright holder Mount Sinai Health System.

This is not to deny that many differences exist among the nervous systems of vertebrates, arthropods, and cephalopods, along with their similarities. Their brains look different and the analogous functional areas do not have the same relative locations in the brains (Figure 3). The similarities still abound, however, so constraints have channeled the emergence of these conscious systems into similar directions (Table 1).

Because the shared features in Table 1 provide real empirical support for Shapiro’s MCT, we will examine several of them to show how strong the constraints can be for the evolutionary convergence of conscious systems. These constrained features are sensory systems, brain organization, mapping, valence neurons, and memory systems. Note as we present them that these features are not unique to conscious systems and animals, but they are necessary for consciousness, and they are much better developed in the conscious animals than in nonconscious animals. So are the *functions* of these features. Thus, they will be informative about consciousness and its constraints.

### Mental Constraints on Conscious Systems

#### Constraints on Sensory Systems

For consciousness to play its role of sensing and mapping the environment in detail, it must have sensory receptors and sensory pathways for all the classes of stimuli: light, mechanical forces, smells, tastes, and temperature. To operate efficiently, these structures must register the location and intensity of each stimulus, and they enhance the contrast between nearby stimuli by a process called lateral inhibition (Shapiro, 2004, Chapter 4). These properties can be seen as constraints that led to convergent evolution because they characterize *all* vertebrates, arthropods, and cephalopods (Hartline et al., 1956; Nahmad-Rohen and Vorobyev, 2019; Kandel et al., 2021).

**Figure 4:**
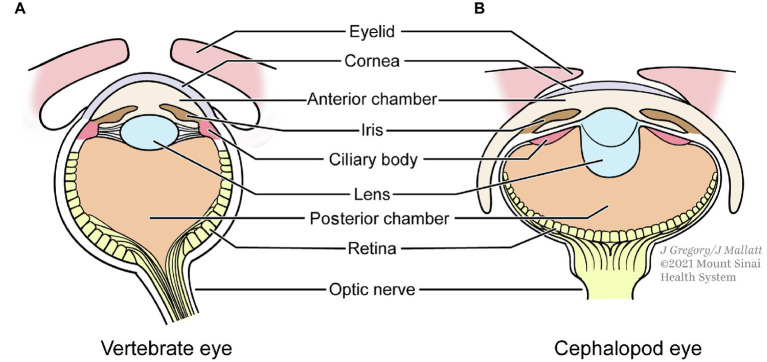
The independently evolved eyes of vertebrates **(A)** and cephalopods **(B)** show strong similarities. **(B)** is redrawn from Hanke and Kelber (2020).

Similarities in the image-forming *eyes* of the three taxa are especially noteworthy. Vertebrates and cephalopods have “simple,” spherical camera eyes that are the most alike, remarkably so considering they evolved independently (Figure 4). Many of the similarities in the lenses, dimensions, and compartments of these two eyes serve to eliminate spherical aberration, a lens problem that blurs the image (Shapiro, 2004, pp. 99–104).

Along with the similarities, the eyes of the conscious clades show some differences. For example, arthropod eyes are not simple but compound, made of many tube-like *ommatidia*. They differ from camera eyes in some significant ways, the retina being convex instead of concave and in having many lenses instead of one. This design sacrifices some visual acuity but is better for detecting movement and it gives the eye a wider field of view.

Despite these differences, compound and simple eyes share many similarities that are demanded by the constraints for image formation: corneas, lenses, and photoreceptor cells. Additionally, the visual pathway from the eye photoreceptors to the visual brain centers is remarkably similar in arthropods and vertebrates (Sanes and Zipursky, 2010). For cephalopods, the visual pathway is far less studied, but it resembles that of arthropods and vertebrates in having especially many levels of successive neurons (Feinberg and Mallatt, 2016; Table 9.2). To summarize this topic, the many similarities between the eyes and visual pathways of the three taxa indicate that only a limited number of structures can produce formed images, favoring Shapiro’s MCT over MRT.

#### Constraints on Brain Organization

A central nervous system contains information-processing neural networks whose neurons are connected by “wires” or “cables” in the form of neuronal axons and dendrites. In such a system, it costs energy to connect and use the wires, so natural selection acts to minimize the cost, especially by minimizing the total length of the wires (Shapiro, 2004, pp. 124–132). Computer simulations show that the best way to minimize cost and maximize fitness is to partition the many neurons into modular groups (local neuronal processing centers), with each *module* having many internal connections but fewer connections to other modules (Simon, 2002). In this way, each module can perform its special processing function and then send a condensed summary out to other parts of the network. A *hierarchical* organization will also emerge, in which the modules have submodules so that each submodule solves a *part* of the module’s processing task (Mengistu et al., 2016). Modularity makes brains more evolutionarily adaptable because “swapping or rearranging maladaptive modules is less costly than rearranging the entire system” (Sporns and Betzel, 2016). Furthermore, having a hierarchy of modules helps to keep a neuronal system in a balanced “critical state,” where the local electrical activity can persist, neither dying out nor spreading uncontrollably through the whole system (Kaiser et al., 2007; Rubinov et al., 2011).

This ideal, modular arrangement takes its highest form in the brains of conscious animals, matching the arrangement we deduced for consciousness (Feinberg and Mallatt, 2019, 2020). We described it as a hierarchical organization with many neural computing modules and networks that are distributed but integrated, for both local functional specialization and coherence among the many parts of the brain. Given this match, these must be constraints that directed the evolutionary emergence of a conscious brain, as happened in vertebrates, arthropods, and cephalopods. Several sources document these traits of increased hierarchy, modularity (brain nuclei and laminae), and fiber connections (tracts and neuropils) in all three of the clades: in the vertebrates (Striedter and Northcutt, 2020), arthropods (plus their nearest relatives the velvet worms: Strausfeld, 2012), and cephalopods (Shigeno et al., 2018; Wang and Ragsdale, 2019). Once more we have uncovered multiple constraints that led to convergent evolution, as Shapiro’s MCT predicted.

#### Constraint of Valence Neurons and Circuits

Having some way to encode value, “good” and “bad,” is a necessity for the affective (~emotional) feelings of consciousness. The existence of value (valence) neurons and circuits is well documented in the brains of vertebrates (Berridge and Kringelbach, 2015; Betley et al., 2015; Namburi et al., 2016; Panksepp, 2016; Tye, 2018), and valence circuits also have been found in arthropods (Felsenberg et al., 2017; Eschbach et al., 2020a,b; Siju et al., 2020). They have not been sought in cephalopods. We should make clear that we are not claiming valence neurons and circuits *explain* good and bad feelings. They are just part of the realizer mechanism that leads to such consciousness.

#### Constraint of Memory Systems

A conscious animal requires a good deal of memory in order to navigate through space using recalled landmarks and in order to learn extensively from past experiences. For these functions of consciousness, memory storage would have to exist in the form of mental representations about the features of this world relevant for a certain species or individual, organized in a more or less episodic way. Thus, we reason that all conscious animals must have relatively large brain regions for memory. This prediction proves true (Figure 3). Vertebrate brains have large memory regions, such as the hippocampus and amygdala (Brodal, 2016), arthropod brains have mushroom bodies for memory (Strausfeld, 2012), and in cephalopod brains, the frontal and vertical lobes participate in sensory memory (Shigeno et al., 2018; Figure 3 in Wang and Ragsdale, 2019). In the three clades, the functional constraints of consciousness independently directed their brains to evolve toward increased memory storage. Once more, constraints led to convergent evolution, as MCT predicts.

### Conclusion of Part 1

Shapiro’s (2004, p. 137–138) book asked whether future empirical research will show if his mental constraint thesis is more valid than the largely unconstrained MRT. Our findings on the convergent evolution of consciousness in vertebrates, arthropods, and cephalopods provide an answer, indicating that MCT is indeed more valid. We accept MCT as better than MRT not only because it fits our own findings but also because unlike standard MRT it incorporates convergent evolution, an important part of evolutionary theory.

Since 2004 Shapiro has developed more ideas on multiple realizability (Shapiro, 2008; Shapiro and Polger, 2012), and he coauthored a book on this subject as Polger and Shapiro (2016). Thus, we must examine that book to see whether these authors’ ideas on MCT have changed and if we still favor them.

## Part 2: Polger and Shapiro on Multiple Realization: Identity Theory After All?

### Points of Agreement

A theme of Polger and Shapiro’s (2016) Multiple Realization Book, henceforth called “P and S,” is that the best explanation of mental processes makes some use of mind-brain identities in a “*modest identity theory*,” meaning that instances of multiple realization are less common than many philosophers assume (pp. 34, 144–145). This turned out to be a logical and direct extension of the authors’ previous ideas on MR. Close reading shows P and S did come to the same conclusion as Shapiro (2004), the conclusion that constraints led to the same mental kinds evolving convergently, with similar neural realizers, in just a few different taxa and that the constraints refute the standard, unconstrained multiple realization thesis (P and S, p. 143).

However, P and S went a step beyond the earlier MCT by calling their new version an identity theory, although one that still allows some mental kinds to be multiply realized (pp. 144–145). Their reason for calling it an identity theory seems to be as follows (p. 143). All brains are complex and more complexity imposes more constraints on the types of neuromechanisms that can perform a given mental function; thus, the complex psychological functions must be realized in “very similar ways” in differently evolved brains due to all the constraints. So far, we can follow their logic, but then P and S apparently equated “very similar ways” with “*identical* ways” to reach their identity theory. That is, they concluded that similarly constrained, convergent solutions are effectively identical solutions. To the contrary, we view “very similar” solutions as nonidentical so we do not consider this—nor the original MCT idea—to be an identity theory. Rather, we see these solutions as highly constrained versions of the multiple realization thesis. Our disagreement, however, may be merely semantic hair-splitting because both we and P and S agree that our two interpretations fall on a spectrum and are close together on this spectrum. That is, there may be no practical difference between our “highly constrained MRT” and their “modest identity theory that allows some MR.”

This means that we and P and S would be in agreement—except for one more thing. They devoted much of their book to arguing against almost every case that has ever been used to support MRT. By contrast, we judge that many of these cases validly support MRT (albeit the constrained version of multiple realizability to which we subscribe).

### Points of Disagreement

The anti-MR cases in question involve (1) neural plasticity, (2) ideas about compensatory differences in mental kinds, and (3) comparing the brains of birds and mammals. Before looking at these cases, however, we must point out that P and S developed valuable and rigorous criteria for judging whether a test case truly indicates MR—an undertaking that has always been difficult and confusing. Here in paraphrased form are their criteria, which they called their Official Recipe (P and S, p. 67):

1. The realized mental kind must be the same in the animals being compared.
2. The realizers must be different.
3. The differences between the realizers must make the kind the same in the two animals.
4. The differences between the realizers cannot be trivial: They cannot be merely the differences one sees within a mental kind.

Although this Official Recipe nicely formalizes the decision process and helps to refute some cases that were wrongly said to support MRT, it cannot always provide certainty. Judgment calls will still remain over whether the kinds are really the same in two individuals (in criterion 1), whether their realizers are really different (in criterion 2), which of the differences are trivial vs. relevant (in criterion 4), etc. The problem of kind-splitting still arises, in which one side says that a purported “kind” is really different subkinds (“split and eliminate:” Aizawa, 2013). For example, P and S (pp. 99–104) used kind-splitting to say that the purported kind, memory, is really many different kinds, such as declarative memory, skills memory, motor learning, and associative learning—to which we retort that all these subtypes of memory involve storage and recall, making them one kind after all-and so on. As another example of the persisting difficulties, if someone claims that two realizers differ (e.g., bird and mammal brains), then it is easy to object by saying they are fundamentally similar. We will apply P and S’s valuable Recipe to various cases and handle such difficulties the best we can.

#### Neural Plasticity and MR

The argument that is most commonly and traditionally used to support MR is neural plasticity, such as when the functions of a damaged part of the cerebral cortex are taken up over time by other parts of the cortex (Block and Fodor, 1972). P and S questioned two prominent experiments that were said to show multiple realization through neural plasticity (pp. 90–98). First was an experiment by Von Melchner et al. (2000), who directed the still-developing visual pathway of newborn ferrets away from the usual, visual, cortex to the differently organized *auditory* cortex and found that the “‘rewired’ ferrets respond as though they perceive stimuli (i.e., light) to be visual rather than auditory.” This would be MR because the ferrets had gained a same kind (vision) through a different route that involved the auditory cortex. However, as P and S point out, tests showed the ferrets’ vision was degraded, with a diminished discriminatory capacity. Therefore, the normal and rewired visual kinds were not the same, the example fails criterion (1) of the Official Recipe, and this is not MR. We agree with P and S’s refutation here. Our disagreements start with the next example.

**Figure 5:**
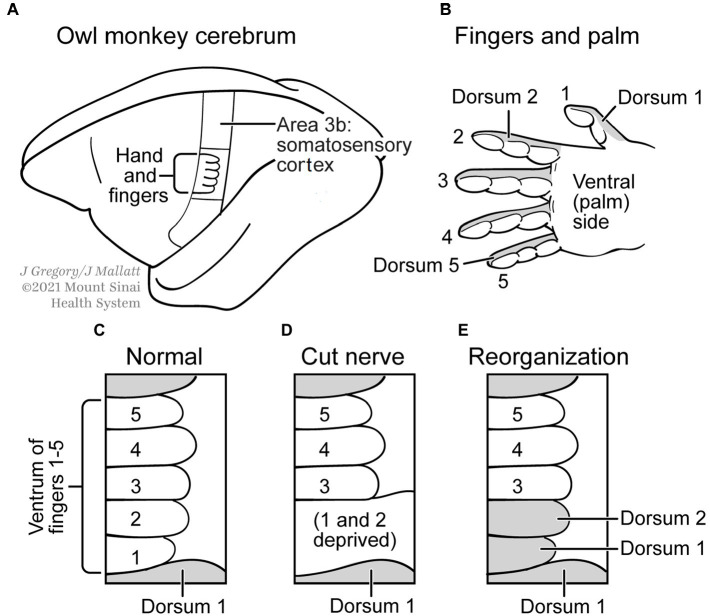
Neural plasticity and multiple realization. **(A)** Cerebral cortex of an owl monkey has an Area S3b, which processes somatosensory (touch) signals from a nerve to the palm side of the hand **(B)**. **(C)** Enlargement of the representation in S3b of a normal monkey, for the five fingers; finger 1 is the thumb and finger 2 is the index finger. **(D)** The representation after the nerve to the first two fingers was cut. **(E)** The areas about a month after the nerve was cut, when some regeneration has occurred. Now the areas for fingers 1 and 2 receive sensory input from the other, *dorsal* (fingernail) side of these digits. Modified from Kaas (1991).

The second plasticity-related example of multiple realization that P and S sought to refute involves the cerebral cortex of the owl monkey, specifically the part of the somatosensory area that represents the fingers for touch sensation (Merzenich et al., 1983a,b; Kaas, 1991). The experiments showed that cutting the nerve to the ventral, fingerprint, side of the first two fingers, which removed all sensory inputs to the cortical representation of this ventral-finger area, was followed by a plastic reorganization of that brain area so it then processed input from the dorsal, fingernail, side (Figure 5). P and S concluded this plasticity does *not* indicate MR, because the ventral-digital area took on a *new* function (of dorsal innervation) and therefore it violated their criterion (1) that says the functional kind must be the same in the two situations, before and after. However, we argue that the experiment does support MR, if we simply shift our perspective over to the dorsal sides of the digits. That is, the sensory processing of this dorsum remains the same functional/mental kind (it is still for touch perception), but now a different cortical-processing area has been added (the area formerly for the ventral sides of the fingers) to the original dorsal processing area. That yields two different realizer areas for the same mental kind, just as MR demands. Therefore, this example of neural plasticity (Figure 5) fits MR.

#### Compensatory Differences and Multiple Realization

Kenneth Aizawa (2013) introduced an argument for multiple realization that he called *multiple realization by compensatory differences* or MRCD. His argument is that when a set of realizing properties contribute jointly to a phenomenon, then changes in some of the properties can be offset by (compensated by) changes in the other properties to keep yielding the original phenomenon. To illustrate this argument, he used equations and formulas for scientific laws as an analogy. Electrical resistance (R) in a wire, for example, is given by R=l.ρ/A, where l is the wire’s length, ρ is the resistivity of the material that makes up the wire, and A is the wire’s cross-sectional area. Thus, the same resistance (kind “R”) results if the area (A) is made smaller and this is counterbalanced by a shorter length or else by replacing the wire with one made of a material with a lower ρ. Other examples are Newton’s second law of motion, F=m.a, where a given force can be attained by a change in mass that balances a change in acceleration, or *vice-versa*, and Ohm’s law for an electrical circuit, I=V/R, where a given current I can be maintained by a change in voltage V that counterbalances a change in resistance R. Aizawa’s MRCD both demonstrates that “there is more than one way to skin a cat” and offers an easily understood reason for this MR thesis.

P and S only briefly addressed the MRCD concept, in a short footnote on page 72 of their book. They argued against MRCD by referring to the R=l.ρ/A example and saying, “In our view, however, these are not multiple realizers of resistance, they are all resisters in the same way.” In other words, they are similarly realized, with the reasoning apparently being that the same three compensating variables (l, ρ, and A) vary along gradients, making them one continuum. P and S seem to be saying that MR requires qualitative, not merely quantitative, differences between its realizers.

For us, this argument against MRCD breaks down when the variables have extremely low or high values, and it breaks down for practical reasons about physical design. Take the F=m.a example. When the particular force is to be achieved by a huge mass that accelerates and moves slowly, such as an earthmover that crawls along, many of the design concerns are about building a massive motor vehicle; but when that same F is to be achieved by rapidly accelerating a tiny object, such as firing a bullet, then the design concerns are much different, mostly about building a handgun. Thus, the mechanisms behind the realizers are qualitatively different and this is still multiple realization. As another example, take Ohm’s law where a particular current I is to be achieved by high voltage V and moderate resistance R. For this, the design can use a powerful lithium battery and an ordinary copper wire. But if the same current I is to be achieved another way—by moderate voltage and low resistance—the design uses an ordinary alkaline battery and a superconducting wire. Again, it is the same realized kind in both cases, they have qualitatively different realizers, and multiple realization (MRCD) is the correct description.

**Figure 6:**
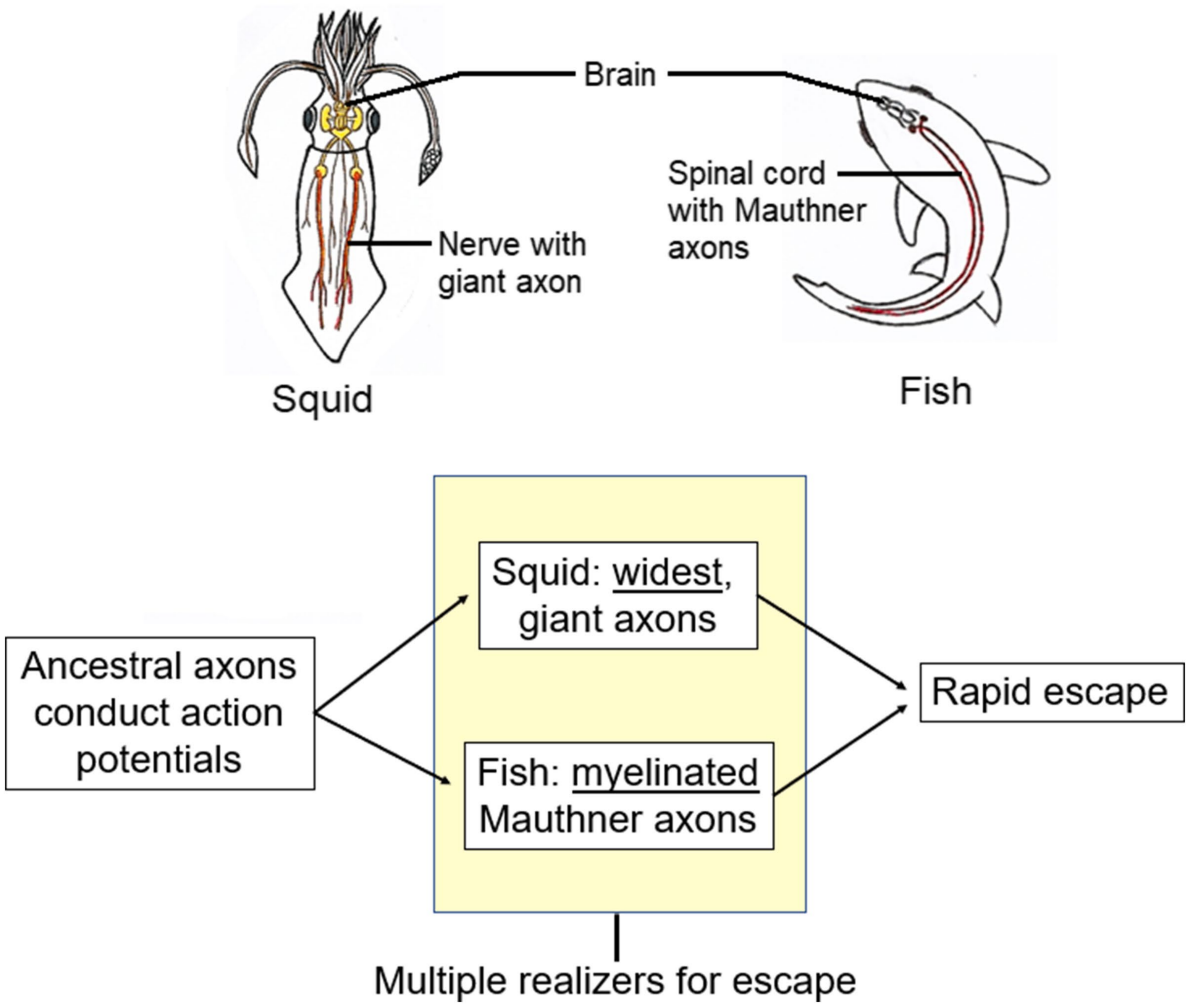
Multiple realizers for signaling rapid escape in squid vs. fish. Squid picture is from Feinberg and Mallatt (2020). Reproduced with the permission of the copyright holder Mount Sinai Health System.

Aizawa’s examples involved simple physical states and he had to assume that compensatory differences also characterize the complex brain states with which classical MR questions deal. This assumption is very difficult to test because of the almost universal lack of knowledge of “exactly what the realizers of psychological states are and how they work” (Aizawa, 2013, p. 79). We can, however, offer an apparent example of a multiply-realized compensatory difference that is, though not of a mental state, at least a brain-signaled behavior. This example is the fast way that squids and fish escape through the water when threatened with danger (Figure 6). Squids use rapidly conducting giant axons to jet-propel away, whereas fish use rapidly conducting Mauthner axons to bend their body then swim off fast (Shapiro, 2004, p 133; Castelfranco and Hartline, 2016). We consider the escape responses of both animals to be the same “kind,” molded by natural selection for survival under the same, threatening, circumstances. Both the types of axons maximize their speeds of impulse conduction but through compensatory differences. For these differences, consider the formula for the propagation velocity (V) of the action potential along the axon that carries the escape signal:


$$V\propto \left(1/C_{m}\right)\;\;\cdot \;\;{\left(d/{\mathrm{4R}}_{m}R_{i}\right)}^{1/2}$$

where C_m_ is the axonal membrane’s capacitance, d is the axon’s diameter, R_m_ is the membrane resistance, and R_i_ is the resistance of the axon’s cytoplasm. The squid giant axon increases the V by maximizing the axon’s diameter d (to 1–1.5mm). The fish axon, by contrast, has a coat of myelin that alters both C_m_ and R_m_ in a way that increases V with only a small increase in d (to 0.04–0.09mm). This is a multiple realization of the function “fast propagation” through a compensatory difference, with squid relying on axonal widening and fish relying more on myelination.

**Figure 7:**
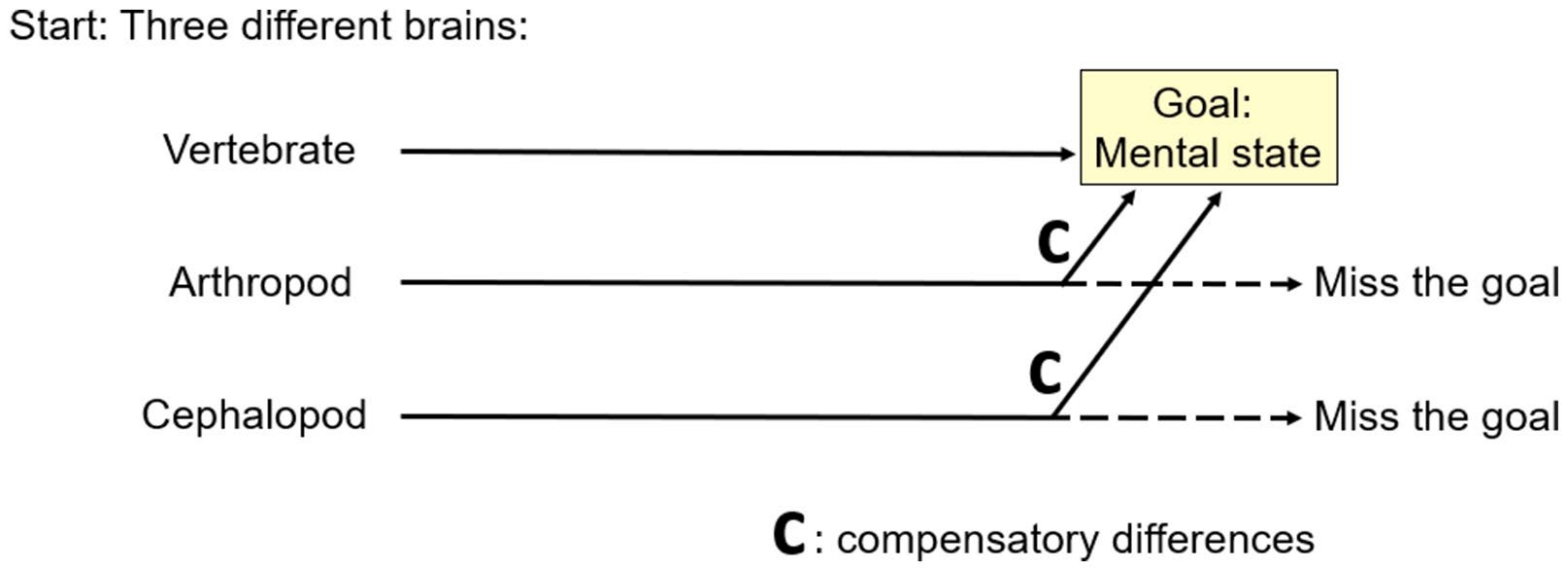
A theoretical reason for multiple realizability by compensatory differences. If different animal lineages start from different places (left), then all but one must evolve compensatory differences if all are to reach a common goal (right).

To us, Aizawa’s MRCD is convincing because, given evolutionary considerations, it seems like it *must* happen. Here is why (Figure 7). As mentioned above, phylogenetic reconstruction indicates the common ancestor of the vertebrates, arthropods, and cephalopods was brainless (Northcutt, 2012) and the immediate ancestors of these three clades had different brains (e.g., Lacalli, 2008; Strausfeld, 2012). Starting from different places demands that MRCD occurred by definition, because otherwise two of the three clades would have missed the goal of the mental state that we argued they do have.

#### Bird and Mammal Pallia

**Figure 8:**
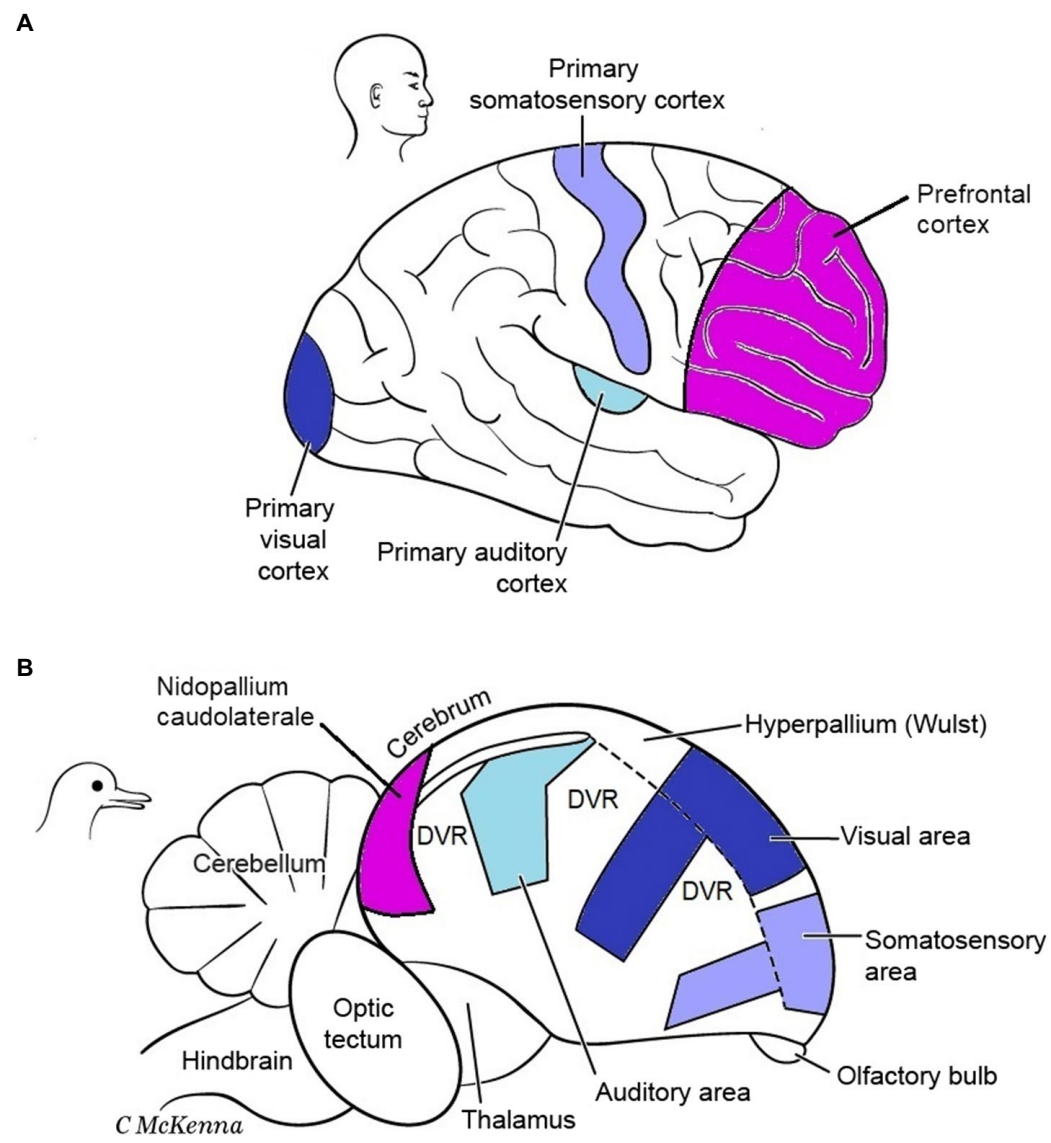
Cerebrums of a mammal **(A)** and bird **(B)**. Functional areas involved in conscious sensory perception and cognition are color-coded. The same functional areas have evolved in different relative locations in these brains. DVR, dorsal ventricular ridge of bird. Modified from Figure 6.9 in Feinberg and Mallatt (2016). The images are reproduced with the permission of the copyright holder Mount Sinai Health System.

P and S examined another test case for whether MR exists, comparing the enlarged cerebral pallia of mammals and birds. In mammals, this brain region is dominated by the cerebral cortex (neocortex), and in birds by functionally equivalent regions called the dorsal ventricular ridge (DVR) plus the cortex-like Wulst (Figure 8). However, the pallium enlarged independently in birds and mammals, from a smaller and more simply organized pallium in their reptile-like common ancestor that lived 350 million years ago (Striedter and Northcutt, 2020). Birds and mammals perform many of the same mental tasks, and it is widely accepted that their convergent pallial expansions permitted the higher mental functions that these taxa share, such as more cognitive abilities, increased memory of objects and events, better problem-solving skills, and improved sensory processing for primary consciousness (Feinberg and Mallatt, 2016; Briscoe and Ragsdale, 2018a; Nieder et al., 2020; Tosches, 2021). Like us, P and S consider these functions to be the same “mental kinds” in birds and mammals because on p. 115 they favorably quoted Karten’s (2013) characterization of these as “virtually identical outcomes.”

Do the neural realizers of these mental kinds differ enough between birds and mammals to indicate MR, or are they similar enough to refute MR instead? As with all MR questions, the detailed neural circuits are not known well enough to answer these questions definitively. However, these are intensely studied brains about which much *is* known, including the basic circuits and many of the differences and similarities (Jarvis et al., 2013; Dugas-Ford and Ragsdale, 2015; Briscoe and Ragsdale, 2018b; Striedter and Northcutt, 2020; Colquitt et al., 2021). Thus, an up-to-date analysis should at least suggest an answer.

**Figure 9:**
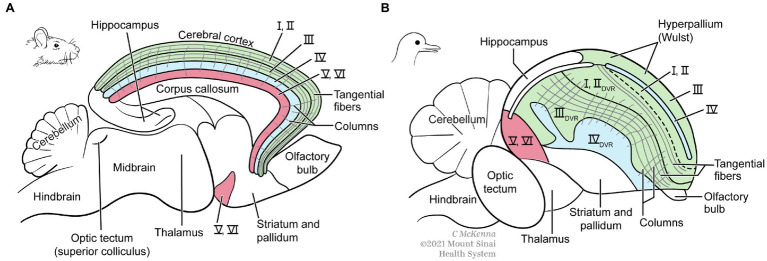
Finer structure of mammal **(A)** and bird **(B)** cerebrums. The mammal brain is cut in half in the sagittal midline. Whereas the mammal neocortex has six thin layers of neurons (I–VI), the analogous structures in birds are thicker and distributed more widely including in the DVR, which mammals lack. Both brains, however, have comparable columns of radially oriented fibers and groups of tangentially oriented fibers. Modified from Figure 6.9 in Feinberg and Mallatt (2016). The images are reproduced with the permission of the copyright holder Mount Sinai Health System.

Gross structural and functional differences seem to support MR (Figure 8). The corresponding functional areas, independently evolved, have different locations in the mammal vs. bird pallia. First, notice the different relative positions of the primary auditory, visual, and somatosensory areas for conscious sensation. Next, notice that the integrative areas for high-level cognition—the prefrontal cortex in mammals and the nidopallium caudolaterale in birds—are in opposite poles of the pallium, front vs. back, respectively (Güntürkün and Bugnyar, 2016). Additionally, mammals have no structure like the DVR of birds. Furthermore, the bird analogues of the six layers of the mammalian cortex are spread widely through the pallium as nuclei (unlayered neuron clusters) or as thick bands (I–VI in Figure 9); this bird state is so unlike the mammal state that it took neurobiologists over a century to even identify the comparable regions (Dugas-Ford et al., 2012; Jarvis et al., 2013). Finally, in embryonic mammals, the cortical layering develops in an outside-in sequence unlike that in birds or any other vertebrate (Tosches et al., 2018; Striedter and Northcutt, 2020, p. 390). So far this looks like very different pallial structures causing similar mental states, apparently an overwhelming argument for MR.

**Figure 10:**
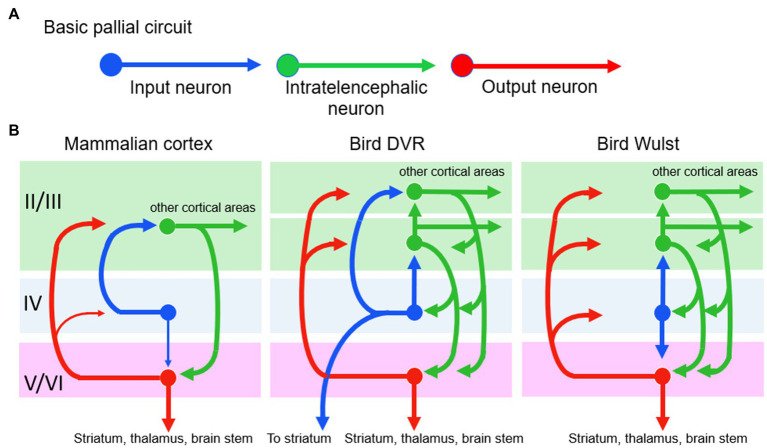
The basic pallial circuit of three kinds of neurons **(A)** is present in mammals and birds. However, differences appear (among the similarities) when the circuit is shown in more detail **(B)**. At left, the numbers II to VI are the numbered layers and structures of Figure 9. Modified from Stacho et al. (2020).

Now let us consider P and S’s argument *against* this being a case of MR. They declare, after Karten, that the basic pallial circuitry is the same in mammals and birds, so that is a causal identity for the identical outcomes, meaning no MR. Figure 10A shows the basic circuit, with an input neuron, an intratelencephalic neuron, and an output neuron. We accept that this three-neuron circuit is homologous in mammals and birds but we say it is too rudimentary to perform the higher mental functions that are considered here. It is basically a three-neuron reflex arc, and reflexes are not higher functions. Even the lamprey, a tiny-brained jawless fish has this basic pallial circuit without any of the higher cognitive functions of mammals and birds (Suryanarayana et al., 2017). No, the bird and mammal circuits would have to be identical at a *higher* level than this to be evidence for identity and against MR.

Therefore, we must look up to the next level of processing (Figure 10B), namely, to the many connections between the three neurons that begin to reflect higher processing. Although this level does show many connectional similarities in birds and mammals, there are notable differences that preclude identity. One difference, shown in the figure, is that in the bird circuits the intratelencephalic neurons (green) send more extensive feedback to the other two neuron types, especially to the input neurons. Another difference is that in the bird DVR the input neurons (blue) project directly to the brain’s striatum, a pre-motor region. These differences could be functionally relevant, especially the striatal projection, because birds make more use of pallial-sensory signals to the striatum than mammals do (Striedter and Northcutt, 2020, p. 318). These signals help the birds to make informed decisions about which motor behaviors to execute in any given context. In summary, we are back to finding differences rather than identity and to finding further support for MR.

Although the evidence so far favors differences and MR, it is important to discuss some additional similarities between the bird and mammal pallia (Wang et al., 2010; Feinberg and Mallatt, 2016; Fernández et al., 2020; Stacho et al., 2020). First, the sensory inputs to both these pallia are arranged according to a body map. Second, the bird pallium contains axon fibers that extend radially and mark out columns that resemble the “cortical columns” of mammals; and third, the bird pallium also contains tangentially running fibers that interconnect distant pallial areas and lie in similar places to such fibers in mammals (Figure 9). We discount these three similarities, however, because Karten and P and S (pp. 115–117) demanded that they be homologous in order to support an identity theory, but they are demonstrably *not* homologous. That is, the similarities are analogues that evolved separately in birds and mammals, as evidenced by the fact that they are absent in today’s reptile relatives of birds—relatives that reveal the pre-bird pallial condition (Striedter and Northcutt, 2020). The reason these similarities evolved independently during brain enlargement in birds and mammals presumably had to do with shared constraints, namely, the need to increase information-processing in more organized and efficient ways, and to save on the cost of axonal wiring (Kaas, 1997, Shapiro, 2004, p. 130). As analogues, they favor the MR interpretation.

We end this section with our formal argument that the “bird-vs.-mammal” example supports MR, contrary to the claim of P and S. According to the Official Recipe, the higher mental kinds in birds and mammals are the same, meeting its criterion (1). The causal realizers show differences (at many levels), meeting criterion (2). The differences between the bird and mammal circuitries could make the mental kinds the same, which would fit criterion (3). And these differences are probably not trivial but relevant to realizing the higher mental processes, which would fit criterion (4).

### More Realizability at Lower Levels?

We have focused on the higher levels of the brain, where we found examples of multiple realizability that had relatively few alternate realizers of mental processes. A possible challenge to this *limited* type of realizability is the possibility of *extensive* realizability at the *lower* levels. That is, as one goes lower in the biological hierarchy (from organ to cells to biomolecules) and encounters more and smaller realizers that could contribute to an overall process, the alternate realizers may become more dissimilar and more numerous. Some examples support this possibility. First, if one goes down to the cell level, one finds a large dissimilarity involving animals called ctenophores. These comb jellies (or sea gooseberries) evolved their nerve cells independently of all the other animals with nervous systems, as revealed by ctenophores’ unique set of synaptic neurotransmitters (Moroz and Kohn, 2016). Second, the submicroscopic action potentials on which neuronal signaling depends can be generated in various, dissimilar ways; e.g., by influxes of Na^+^ in animals vs. influxes of Ca^2+^ in plant cells (Mallatt et al., 2021). Third, down at the intracellular level, many alternate enzymatic pathways can perform the same metabolic role through multiple realizability, a form of redundancy that aids cellular survival (Wagner, 2014, Chapter 6). A fourth example of more MR at lower levels goes down to the genes: A number of studies have found that different genes can account for the same phenotypic adaptation in different organisms (Natarajan et al., 2016; James et al., 2020; Figure 1 in Pyenson and Marraffini, 2020; Colella et al., 2021). While these are all valid examples of MR to add to our growing list, do they really show that MR is more common at lower levels? Do they take us back to standard MRT, with its “very many” possible realizers?

Probably not, because many counterexamples show *identity* at the lower levels. First, some genetic studies of the parallel evolution of phenotypes reveal “identical mutations fixed independently” (Sackton and Clark, 2019). Second, numerous other lower-level features are the same in all animals. These universally conserved features include: the presence of epithelium and connective tissues; the same, eukaryote cell type with the same suite of cellular organelles; the same 64 codons for the genetic code; and the same four nucleotides of DNA (A, C, G, and T; Ruppert et al., 2004). In these lower-level examples, there is far less variability than we found among brain regions at the higher levels (Figure 3), throwing doubt on the entire claim for more realizers at the lower levels. Where they are rigidly conserved, the lower-level features seem to reflect strong stabilizing selection for survival (e.g., epithelial sheets are the most effective tissues for borders in animal bodies; animal cells without all the typical organelles would be less fit). Therefore, whether or not the instances of multiple realization are more numerous at lower levels of the biological hierarchy, they remain limited by survival constraints. Such constraints operate at every level of biological hierarchies and the multiple-constraint part of Shapiro’s thesis still holds true.

### Conclusion of Part 2

We agree substantially with the ideas of P and S, but not completely. The disagreements are that we accepted more examples of MR than they did (e.g., neural plasticity, bird-mammal pallia, and alternate enzymatic pathways for cell metabolism) and we accept Aizawa’s (2013) proposal that compensatory differences generate multiple realizability. Thus, we say that P and S went too far in arguing against MR. We found that there can be more ways to achieve a mental state than just Shapiro’s “handful” (though still fewer ways than standard MRT claims). It should be easy to reconcile our disagreements with P and S because they explicitly designed their modest identity theory to allow more instances of true MR, as long as this also allows some substantial instances of identity (p. 34). A central point of agreement is that both we and they recognize the importance of convergent constraints in limiting the number of realizations, which the standard MRT—with its almost numberless realizations—does not.

## Conclusion

Our consideration of animal evolution reveals that the emergence of consciousness proceeded under many constraints and therefore involved strong evolutionary convergences between vertebrates, arthropods, and cephalopods (Table 1), as well as between birds and mammals (Figures 8–10). This emergence proceeded along the multiple routes of a highly constrained multiple realizability. Table 3 provides a summary by comparing our present conclusions with the standard MRT, Shapiro’s (2004) mental constraint thesis, and Polger and Shapiro’s (2016) modest identity theory.

**Table 3:** Comparison of the theories presented in this paper, on the realizability of mental states in different taxa.

Standard Multiple Realizability (Bickle, 2020)	Mental Constraint of Shapiro (2004): MCT	Modest Identity of Polger and Shapiro (2016): MIT	Our Constrained Multiple Realizability
1. Many realizers for each mental kind (thousands or more)	1. Few realizers for each mental kind (handful)	1–3. Same as for MCT, and rejects most of the classic examples of multiple realization	1. Few realizers for each mental kind (but can be more than a handful)
2. Constraints are not recognized	2. Constraints are common		2. Constraints are common
3. Convergent evolution is not recognized	3. Convergent evolution is important		3. Convergent evolution is important
4. No identity of mind and brain.	4. Mind-brain identity is not refuted by any multiple realizability	4. Promotes a kind of mind-brain identity by saying strong similarities in brain mechanisms are effectively identities; such identities are common, but MIT tolerates at least some instances of multiply-realized non-identities	4. Strong similarities are not identities, so we recognize more examples of true multiple realization than MIT does. Ours is more of a highly constrained version of MRT than an identity theory

## Data Availability Statement

The original contributions presented in the study are included in the article. Further inquiries can be directed to the corresponding author.

## Author Contributions

JM analyzed the theories and wrote most of the manuscript. TF provided much of the information on consciousness and emergence. All authors contributed to the article and approved the submitted version.

## Conflict of Interest

The authors declare that the research was conducted in the absence of any commercial or financial relationships that could be construed as a potential conflict of interest.

## Publisher’s Note

All claims expressed in this article are solely those of the authors and do not necessarily represent those of their affiliated organizations, or those of the publisher, the editors and the reviewers. Any product that may be evaluated in this article, or claim that may be made by its manufacturer, is not guaranteed or endorsed by the publisher.

## Footnotes

^1^The related term, multiple realization, is also used. “Multiple realizability” refers to all the possible physical causes (including imaginary ones), whereas “multiple realization” refers only to the known physical candidates like neurons and brains (Bickle, 2020). We will not make this realizability-realization distinction, however, because the literature we are reviewing seldom distinguishes the terms.

^2^Although “multiple realizability” was originally applied only to mental states, now this term is also being applied to the functional states of other complex physical systems (Ellis, 2012). These multiply-realized states range from convection currents in liquids (Bishop and Silberstein, 2019), to protein biochemical states (Tahko, 2020), to the elasticity of different polymers (McLeish, 2019), to transitions at a critical point in fluids and ferromagnets (Blundell, 2019), and to electrical wires and more (Aizawa, 2013).

^3^Our reasoning behind these two assumptions is: (1) it is logical to say that if an animal exerts the energy to build the detailed and mapped representations, then it will use these representations as mental reference images to help it move and operate in the real world; (2) complex operant learning seems to provide two-part evidence that an animal feels emotions, both the initial attraction to a reward and then recalling the reward-feeling that motivates the learned behavior (Mallatt et al., 2021).
